# Supplementary material for: PHFinder: assisted detection of point heteroplasmy in Sanger sequencing chromatograms
Source: PeerJ. 2023 Sep 19;11:e16028. doi: 10.7717/peerj.16028 (PMC10516101; doi:10.7717/peerj.16028)
Supplement: Supplemental Information 2 [file peerj-11-16028-s002.pdf]

Supporting Information for **PHFinder**: Assisted detection of point heteroplasmy in Sanger sequencing chromatograms.

Authors: Marcos Suárez Menéndez, Vania Rivera-León, Jooke Robbins, Martine Bérubé & Per J. Palsbøll.

Corresponding: m.suarez.menendez@rug.nl

Table S1. Samples with known heteroplasmies in the dataset, AB1 files, position of the heteroplasmy in the reference sequence (NC_006927.1) and MR at the heteroplasmic nucleotide site.

Sample ID	AB1 file	Position	Main ratio
MN001	5092_6.ab1	258	88
MN001	5095_14.ab1	258	73
MN001	5097_4.ab1	258	76
MN010	5985_38.ab1	258	40
MN014	5982_53.ab1	143	58
MN020	5981_17.ab1	143	21
MN026	5500_3.ab1	82	32
MN027	5497_38.ab1	82	86
MN043	5504_9.ab1	235	63
MN033	5515_54.ab1	82	24
MN035	5512_12.ab1	258	68
MN013	5507_2.ab1	82	41
MN056	4731_18.ab1	281	53
MN060	4751_17.ab1	258	47
MN097	4751_15.ab1	282	59
MN097	4744_38.ab1	282	46
MN061	4744_32.ab1	82	21
MN062	4729_37.ab1	143	26
MN063	4661_65.ab1	281	75
MN063	4666_19.ab1	281	76
MN063	5101_11.ab1	281	78
MN064	4661_53.ab1	258	29
MN065	4734_3.ab1	143	21
MN066	4661_37.ab1	82	31

MN066	5101_3.ab1	82	33
MN069	4661_8.ab1	281	46
MN071	4661_1.ab1	258	49
MN071	4666_17.ab1	258	58
MN058	4655_6.ab1	258	60
MN058	4667_80.ab1	258	59
MN081	5509_19.ab1	258	73
MN085	4948_82.ab1	82	29
MN086	4948_79.ab1	143	73
MN086	5311_26.ab1	143	64
MN088	4948_62.ab1	143	27
MN088	5138_89.ab1	143	20
MN090	4626_23.ab1	258	70
MN095	5138_28.ab1	258	41
MN095	4954_29.ab1	258	74
MN017	4598_77.ab1	235	27
MN017	4667_38.ab1	235	18
MN098	4598_53.ab1	258	48
MN098	4667_36.ab1	258	40

Notes: Positions based on the region analysed; position 1 corresponds to position 15,490 in the reference NC_006927.1 (NCBI Reference Sequence) from Sasaki et al. (2005).

Table S2. Summary of the results for each of the 64 sets of index threshold values.

Parameters	HP_AB1	HP_Samples	False_Positives	Analysed_Samples	Analysed_AB1	Open_AB1
MR15-SR0.2-AQ30	21	16	1	100	143	22
MR15-SR0.2-AQ40	20	15	1	88	115	21
MR15-SR0.2-AQ50	14	12	0	68	79	14
MR15-SR0.2-AQ60	3	3	0	37	15	3
MR15-SR0.3-AQ30	31	22	9	100	143	38
MR15-SR0.3-AQ40	30	21	5	88	115	34
MR15-SR0.3-AQ50	21	15	2	68	79	23
MR15-SR0.3-AQ60	4	4	0	37	15	4
MR15-SR0.4-AQ30	39	28	38	100	143	66
MR15-SR0.4-AQ40	38	27	23	88	115	52
MR15-SR0.4-AQ50	29	21	13	68	79	36
MR15-SR0.4-AQ60	7	7	0	37	15	7
MR15-SR0.5-AQ30	41	30	113	100	143	94
MR15-SR0.5-AQ40	39	28	61	88	115	67
MR15-SR0.5-AQ50	30	22	25	68	79	42
MR15-SR0.5-AQ60	7	7	2	37	15	8
MR20-SR0.2-AQ30	21	16	1	100	143	22
MR20-SR0.2-AQ40	20	15	1	88	115	21
MR20-SR0.2-AQ50	14	12	0	68	79	14
MR20-SR0.2-AQ60	3	3	0	37	15	3
MR20-SR0.3-AQ30	30	22	7	100	143	35
MR20-SR0.3-AQ40	29	21	4	88	115	32
MR20-SR0.3-AQ50	20	15	1	68	79	21
MR20-SR0.3-AQ60	4	4	0	37	15	4
MR20-SR0.4-AQ30	38	28	18	100	143	52
MR20-SR0.4-AQ40	37	27	8	88	115	43
MR20-SR0.4-AQ50	28	21	4	68	79	32
MR20-SR0.4-AQ60	7	7	0	37	15	7
MR20-SR0.5-AQ30	40	30	66	100	143	78
MR20-SR0.5-AQ40	38	28	31	88	115	57
MR20-SR0.5-AQ50	29	22	11	68	79	37
MR20-SR0.5-AQ60	7	7	1	37	15	8
MR25-SR0.2-AQ30	20	15	1	100	143	21
MR25-SR0.2-AQ40	19	14	1	88	115	20
MR25-SR0.2-AQ50	13	11	0	68	79	13
MR25-SR0.2-AQ60	3	3	0	37	15	3

MR25-SR0.3-AQ30	28	21	5	100	143	31
MR25-SR0.3-AQ40	27	20	3	88	115	29
MR25-SR0.3-AQ50	18	14	0	68	79	18
MR25-SR0.3-AQ60	3	3	0	37	15	3
MR25-SR0.4-AQ30	34	25	9	100	143	40
MR25-SR0.4-AQ40	33	24	4	88	115	35
MR25-SR0.4-AQ50	24	18	0	68	79	24
MR25-SR0.4-AQ60	5	5	0	37	15	5
MR25-SR0.5-AQ30	35	26	40	100	143	57
MR25-SR0.5-AQ40	34	25	18	88	115	44
MR25-SR0.5-AQ50	25	19	6	68	79	30
MR25-SR0.5-AQ60	5	5	1	37	15	6
MR30-SR0.2-AQ30	19	14	1	100	143	20
MR30-SR0.2-AQ40	18	13	1	88	115	19
MR30-SR0.2-AQ50	12	10	0	68	79	12
MR30-SR0.2-AQ60	2	2	0	37	15	2
MR30-SR0.3-AQ30	25	18	3	100	143	28
MR30-SR0.3-AQ40	24	17	2	88	115	26
MR30-SR0.3-AQ50	15	11	0	68	79	15
MR30-SR0.3-AQ60	2	2	0	37	15	2
MR30-SR0.4-AQ30	29	20	5	100	143	33
MR30-SR0.4-AQ40	28	19	3	88	115	30
MR30-SR0.4-AQ50	19	13	0	68	79	19
MR30-SR0.4-AQ60	3	3	0	37	15	3
MR30-SR0.5-AQ30	30	21	25	100	143	46
MR30-SR0.5-AQ40	29	20	15	88	115	37
MR30-SR0.5-AQ50	20	14	4	68	79	23
MR30-SR0.5-AQ60	3	3	0	37	15	3

Notes: HP_AB1: Number of AB1 files with an heteroplasmy detected. HP_Samples: Number of heteroplasmic samples detected. False_Positives: Number of false positives detected. Analysed_Samples: Number of samples analysed by PHFinder. Open_AB1: Number of AB1 that would need to be manually checked.
